# The Joule Heating Effect of a Foldable and Cuttable Sheet Made of SWCNT/ANF Composite

**DOI:** 10.3390/nano12162780

**Published:** 2022-08-13

**Authors:** Min Ye Koo, Gyo Woo Lee

**Affiliations:** Division of Mechanical Design Engineering, Jeonbuk National University, 567 Baekje-daero, Deokjin-gu, Jeonju 54896, Korea

**Keywords:** single-walled carbon nanotubes (SWCNTs), aramid nanofibers (ANFs), thermal stability, electrical property, composite sheet, Joule heating

## Abstract

A foldable and cuttable sheet heater was fabricated using single-walled carbon nanotubes (SWCNTs) and aramid nanofibers (ANFs). SWCNTs are particularly well suited for Joule heating based on their high thermal stability, electrical properties, high current density, and aspect ratio. When the SWCNT/ANF composite reaches a high temperature during Joule heating, ANFs will endure this temperature due to their impressive thermal stability, derived from aramid fibers. With the aim of achieving a synergistic effect between the SWCNTs and ANFs, 0–100 wt% SWCNT/ANF composite sheets were fabricated by tip-type sonication and vacuum filtration. After assessing the thermal stability and electrical properties of the composite sheets, the Joule heating effect was analyzed. TGA showed that our sheet had high thermal stability in an air condition up to around 500 °C. The electrical conductivity of the composite sheet was improved as the amount of SWCNT added rose to 790.0 and 747.5 S/cm in the 75 and 100_SWCNTs/ANF, respectively. The maximum heating temperature, up to 280 °C, reached by Joule heating was measured as a function of SWCNT content and input voltage, and the relationship among SWCNT content, input voltage, heating temperature, and electric power was described. Mechanical properties were also measured in a temperature range similar to the heating temperature of 300 °C reached by Joule heating. Ultimately, we obtained a foldable and cuttable composite sheet with a stretchable structure, capable of being molded into a variety of shapes. This energy-efficient material can potentially be employed in any device in which a heater is required to deliver high temperatures.

## 1. Introduction

Carbon nanotubes (CNTs) are today regarded as the most important one-dimensional (1D) nanomaterial for their attractive electrical, thermal, and mechanical properties, and their associated applications [1,2,3,4,5,6,7,8]. Single-walled carbon nanotubes (SWCNTs) take the form of a single graphite sheet rolled into a tube. This material, with its unique structure, is particularly well suited for Joule heating based on its high current density, thermal stability, aspect ratio, etc. [9,10,11,12,13,14,15,16,17,18,19]. These characteristics allow Joule heating to convert electrical energy into thermal energy, and Joule heating has accordingly attracted a great deal of attention. It is no exaggeration to say that Joule heating is used in almost every product that requires heat, including radiative heaters, hot plates, soldering irons, heating mats, and de-icing devices. Metal is typically used as a Joule heating material, although, today, many researchers are attempting to use CNT-reinforced composites as an alternative, as these offer a shorter heat-up time, more uniform temperature distribution and flexible structure, a lighter weight, etc. [20,21,22,23]. If a sheet heater is flexible, cuttable, and foldable, it can be used in a variety of shapes of different sizes. Wherever heat is required, it can be efficiently transferred with reduced heat loss.

Joule heating appears when an electrical current flows along a CNT [24]. CNTs have a high current density of approximately 10^9^ A cm^−2^, meaning that CNTs have a superior Joule heating effect when compared to other materials [10]. Notably, the current density of copper, which is a commonly used metal for Joule heating, is 10^6^ A cm^−2^ [24]. Deshpande et al. observed the spatial temperature of SWCNTs under Joule heating conditions using Raman spectroscopy [11]. When 1.2 V was applied to the SWCNT, the resulting temperature reached approximately 400 °C across a 5 μm length.

If CNTs are used as fillers in composites, the matrix should possess superior thermal stability. One representative polymer with excellent thermal stability is poly (m-phenylene isophthalamide, PMIA), also known as aramid fiber [25,26,27]. Jeong et al. fabricated multi-walled carbon nanotube (MWCNT)-reinforced poly (m-phenylene isophthalamide) (m-aramid) composite films using solution casting, in which they dissolved m-aramid fibers in solvent and salt for wrapping MWCNTs [28]. When they investigated the electric heating elements with 10 wt% MWCNTs reinforced in the composite films, a maximum temperature of around 270 °C was achieved at a voltage of 12 V. Yang et al. obtained 40 wt% reinforced CNT/PMIA (poly (m-phenylene isophthalamide)) composite paper with a maximum temperature of 270 °C under a voltage of around 15 V [29]. This was prepared by a fabrication method in which CNTs were reinforced with PMIA fiber and PMIA fibrid slurry. Wang et al. also fabricated electric heating paper based on carboxylic carbon nanotubes reinforced with PMIA composite [30]. In their study, 30 wt% carboxylic CNTs were dispersed into m-aramid fibrid solutions. These composite papers reached 242 °C when powered with an input voltage of 25 V.

A careful review of these studies suggests that Joule heating is enhanced as the CNT content and applied voltage increases [28,29,30,31,32,33,34]. We therefore expect that when the CNTs are included as much as possible within composites, superior Joule heating effects will be exhibited. A further conclusion to be drawn from these studies is that when aramid fibers are used as the matrix for composites, the Joule heating ability of CNTs depends on how the aramid fibers are modified. Based on these reasons, SWCNT composites can be designed to enable efficient energy conversion from electrical energy to thermal energy, accounting for the allowable electric power, input voltage, the electrical conductivity of the composite material, and the heating temperature during Joule heating.

Aramid nanofibers (ANFs) are a 1D material decomposed from aramid fibers at a micro scale using the top-down method [35]. Various studies of ANFs have suggested that the high thermal stability and mechanical properties of aramid fibers are maintained with almost no loss as a result of this process [36,37,38,39,40]. ANFs are abundant in polar functional groups, including carboxylic acid, carbonyl, and hydroxyl groups, on their surfaces [38]. In short, a synergistic effect between the SWCNTs and the ANFs is to be expected when a new composite sheet that uses these 1D materials is fabricated.

In this study, foldable and cuttable sheet heaters were successfully fabricated using ANF composite sheets reinforced with SWCNTs. Stretchable structures, which are foldable and cuttable, suggest a higher level of possibility than those of flexible and twisted sheet heaters. Here, 0–100 wt% SWCNT/ANF composite sheets were prepared by tip-type sonication and vacuum filtration. The Joule heating effect was evaluated by analyzing the electrical properties and thermal stability of the composite sheet. The heating temperature reached during Joule heating was observed with a thermal imaging camera. This temperature was measured as a function of SWCNT content and input voltage, and the relationship between electric power and electrical conductivity was analyzed based on these results. Finally, mechanical properties were measured in a temperature range similar to that of the heating temperature reached during Joule heating. Ultimately, we obtained a foldable and cuttable SWCNT/ANF composite sheet with a stretchable structure capable of Joule heating while maintaining its mechanical properties.

## 2. Materials and Methods

### 2.1. Materials

SWCNTs (Tuball) were obtained from OCSiAl Inc. (Novosibirsk, Russia). The quality of SWCNTs was G/D ratio >90. The aramid fibers (Heracron) used in this work were purchased from Kolon Inc. Dimethyl sulfoxide (DMSO) and potassium hydroxide (KOH) were purchased from Daejung Chemical & Metals Inc (Siheung-si, Korea).

### 2.2. Fabrications

**Figure 1:**
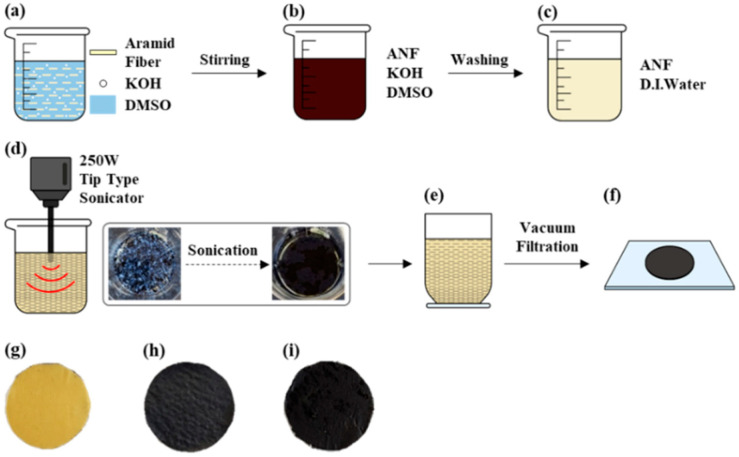
Illustrations and images concerning the fabrication process of SWCNT/ANF composite sheets. (**a**) Aramid fibers/DMSO/KOH solution, (**b**) ANF solution (ANFs/DMSO/KOH) of dark red solution, (**c**) ANF/DI water of light yellow solution, (**d**) SWCNT and ANF/DI water solution before and after tip-type sonication, (**e**) vacuum filtration, and (**f**) dispersion solutions by sonication were made into composite sheets through vacuum filtration. (**g**) 0_SWCNT/ANF, (**h**) 50_SWCNT/ANF, and (**i**) 100_SWCNT/ANF. The platinum sputter coating was executed for all samples.

The ANF solution was prepared using the method described by Kotov et al. [35]. First, 1 g of aramid fibers (5 mm long) and 1.5 g KOH were added into 500 mL of DMSO (Figure 1a). The ANF solution was stirred for 1 week at room temperature, until a dark red solution was obtained. Then, 500 mL deionized (DI) water was poured into the ANF solution, which was heated at 80 °C for 2 h to extract the ANFs. The precipitated ANFs were subsequently washed several times with DI water using vacuum filtration to remove the KOH and DMSO, until a pH of 7 was reached. Up to this process, we obtained the dispersed ANFs in DI water, denoted as ANF/DI water solution. The ANF/DI water solution (1 g/L) was processed using a tip-type sonicator (VCX-500, Sonics&Materials Inc., Newtown, Connecticut, USA) for 30 min and bath type sonicator for 10 min to completely disperse the ANFs. The dark red solution depicted in Figure 1b contains ANFs, KOH, and DMSO, while the light yellow solution is ANFs dispersed in DI water (ANF/DI water, Figure 1c).

SWCNT/ANF composite films were fabricated by vacuum filtration with PVDF membrane filters (47 mm diameter, 0.2 μm, Millipore Inc., Burlington, Massachusetts, USA) and as-received SWCNTs dispersed in ANF/DI water solution. The SWCNTs in the mixture were dispersed using a tip-type sonicator for 60 min, which included 2 min executions and 2 min rests. Figure 1d shows the SWCNT and ANF/DI water solution before and after tip-type sonicating. The SWCNTs dispersed in the ANF/DI water were filtrated by vacuum filtration to remove the DI water (Figure 1e). DI water was then completely removed using a convection oven overnight at 80 °C (Figure 1f). Composite sheets in which the weight fractions of the SWCNTs were 0, 25, 50, 75, and 100 wt% were prepared under identical conditions. Samples were named 0, 25, 50, 75, and 100_SWCNT/ANF, based on the SWCNT weight fractions. Additional details pertaining to the 0, 50, and 100_SWCNT/ANF appear in Figure 1g–i.

### 2.3. Measurements

The surfaces and cross-sections of the sheets were observed using a field-emission scanning electron microscope (FE-SEM, SU-70, HITACHI Inc., Tokyo, Japan) at a voltage of 10 kV. Cross-sections of each sample were cut with an ion-milling system (IMS, IM5000 HITACHI Inc., Tokyo, Japan). The platinum sputter coating was executed for samples. Infrared microscopy was used to record the FT-IR (Fourier Transform Infrared Spectrometer, Frontier, Perkin Elmer Inc.). The structure of each sample was investigated with X-ray diffraction (XRD, D/MAX 2500, Rigaku Instrument Inc., Tokyo, Japan) using Cu Ka radiation, with a 0.1541 nm wavelength, 40 kV tube voltage, 100 mA current, 4°/min scan speed, and a scan range (2θ) of between 15° and 30°. The thermal stability of each sample was confirmed using thermal gravimetric analysis (TGA, Q600, TA Instruments, Waters Inc., New Castle, Delaware, USA). The processing temperature was increased to 800 °C from room temperature at a heating rate of 10 °C/min with an air flow rate of 50 sccm. Electrical conductivity was measured using the 4-point probe method (RSD-1G, Dasol Eng Inc., Cheongju, Korea). To create our Joule heating systems, a voltage was applied to each sample using a DC power supply (Agilent U8002A, Keysight Technologies Inc., Santa Rosa, California, USA), and the resulting temperature was detected with a thermal imaging camera (Testo 870, Testo Inc., Sparta, New Jersey, USA). Dynamic mechanical analysis (DMA, Q800, TA Instruments, Waters Inc., New Castle, Delaware, USA) tests were executed within a temperature range of 50 and 300 °C at a heating rate of 5 °C/min in tensile mode. The strain amplitude was 0.2% with a frequency of 1 Hz.

## 3. Results

### 3.1. Morphology

**Figure 2:**
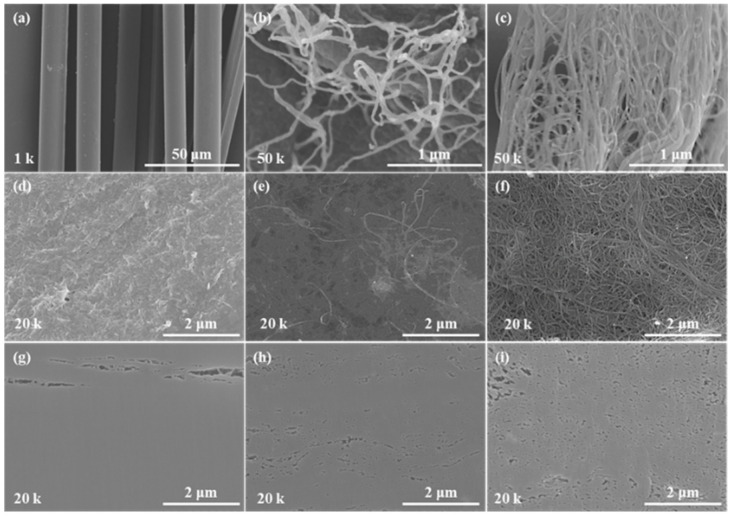
FE-SEM image of (**a**) as-received aramid fiber, (**b**) decomposed ANFs from aramid fiber, and (**c**) as-received SWCNT powder. Surfaces of (**d**) 0_SWCNTs/ANF, (**e**) 50_SWCNTs/ANF, and (**f**) 100_SWCNTs/ANF. Cross-sections of (**g**) 0_SWCNTs/ANF, (**h**) 50_SWCNTs/ANF, and (**i**) 100_SWCNTs/ANF.

FE-SEM images at various magnifications are shown in Figure 2. As-received aramid fibers approximately 10 μm in diameter are presented in Figure 2a. In Figure 2b, we can observe that the ANFs are successfully split from the aramid fibers. Figure 2c shows the SWCNTs as received, with SWCNT bundles in an entangled state. While the diameters of the SWCNTs used in this experiment were around 2 nm [41], SWCNTs exist in bundles due to van der Waals forces. The diameter of a single strand of ANF is approximately 40 nm, whereas a bundle of SWCNTs are approximately 40 nm in diameter. Figure 2d–f are the surfaces and Figure 2g–i are the cross-sections of the composite sheet. Figure 2d,g show 0_SWCNT/ANF, which was composed of only ANFs. Although long-shaped voids were found in the cross-section of the sheet, most of the ANFs appeared to be densely interconnected. Figure 2e,h are 50_SWCNT/ANF. Unfortunately, SWCNTs and ANFs were not clearly distinguished by the FE-SEM in this composite sheet. Figure 2f,i show the 100_SWCNT/ANF, which was composed of only SWCNTs. SWCNT bundles are randomly entangled to form the SWCNT sheet, with many small-sized voids. None of the long-shaped voids observed in 0_SWCNT/ANF were observed in the 50_SWCNT/ANF, while small voids were partially found in 100_SWCNT/ANF, i.e., 50_SWCNT/ANF has an intermediate morphology between 0 and 100_SWCNT/ANF.

Excluding the long-shaped void at the top of the image in Figure 2g, 0_SWCNT/ANF were successfully assembled into microscale sheets, as shown in Figure 2d,g as a result of the abundant functional groups of the ANFs [38]. In contrast, the 100_SWCNT/ANF had a lot of unnecessary voids because SWCNTs were randomly entangled (Figure 2f,i). When dispersed SWCNTs were placed among the ANFs in ANF/DI water, the ANFs were able to prevent the random entanglement of SWCNTs. Accordingly, the number of small voids was significantly lower in 50_SWCNT/ANF.

**Figure 3:**
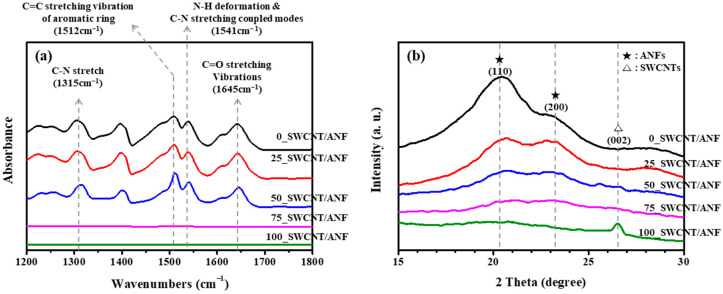
Characteristic curves of SWCNT/ANF composite sheets with varying SWCNT content. (**a**) IR absorbance spectra of aramid fibers and ANFs with wavenumbers between 1200 and 1800 cm^−1^, and (**b**) XRD spectra.

FT-IR and XRD spectra were used to determine the molecular structures of the SWCNT/ANF composite sheets. Since the 0_SWCNT/ANF sample consisted of only ANFs, the representative peaks of aramid fibers appear in Figure 3a (and are indicated in Figure 3b). These peaks faded as the SWCNT content increased to become higher than that of ANF [35,36,37]. In the XRD spectra (Figure 3b), the characteristic peaks of 0_SWCNT/ANF appeared at a 2θ value of 20.3° (110) and 23.1° (200), which are also the characteristic peaks of aramid fibers [35,36,37]. The intensity of these ANF peaks weakened slightly as the SWCNT content increased. We observed in the 100_SWCNT/ANF sample a characteristic peak of SWCNT at approximately 26.5° (002) [40].

### 3.2. Thermal Stability

**Figure 4:**
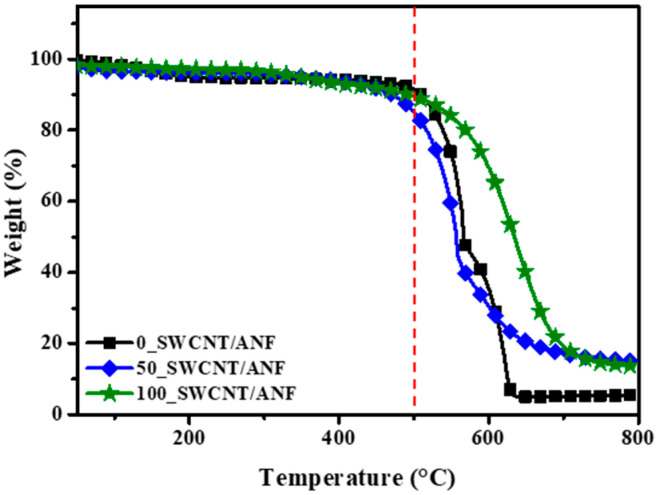
TGA plots of 0, 50, and 100_SWCNT/ANF samples. The red dotted line indicates 500 °C.

TGA was also performed to confirm the thermal stability of the SWCNT/ANF composite sheets. The processing temperature was increased from 50 to 800 °C at a rate of 10 °C/min with an air flow rate of 50 sccm. The measured results of the 0, 50, 100_SWCNT/ANF samples are shown in Figure 4 as a function of temperature. Typically, these materials begin to be decomposed by the oxygen in the air in high heat conditions. Each of the samples that we observed began to decompose at around 470–500 °C. The red vertical line in Figure 4 indicates 500 °C. Although we noted a difference in the burning rate, it could be mentioned that they have high thermal stability in an air condition up to around 500 °C.

### 3.3. Electrical Conductivity

**Figure 5:**
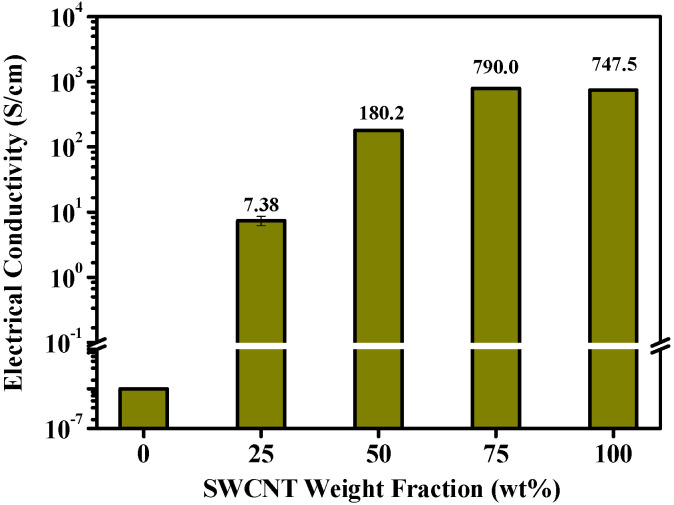
Electrical conductivity of SWCNT/ANF composite sheets according to SWCNT weight fraction.

The excellent electrical properties of SWCNTs suggest that the electrical conductivity of SWCNT/ANF composites will consistently increase as the SWCNT concentration rises, despite the fact that ANFs are electric insulators. As we anticipated, the electrical conductivity of the composite sheet was observed to have improved with the addition of SWCNTs (Figure 5). The 0_SWCNTs/ANF acted as an insulator. The average and standard deviations were calculated by measuring five points per sample of three specimens. The averaged electrical conductivities of each specimen were 7.38, 180.2, 790.0, and 747.5 S/cm for the 25, 50, 75, and 100_SWCNTs/ANFs, respectively. Our tests also confirmed that SWCNTs were uniformly dispersed with a low standard deviation, which was 1.22, 2.14, 12.23, and 13.13 for the 25, 50, 75, and 100_SWCNTs/ANFs, respectively. Notably, the 75 and 100_SWCNT/ANF showed similar electrical conductivity results.

In the 100_SWCNT/ANF, an undesirable air space existed between the SWCNT bundles, as shown in Figure 2f,i. Depending on how this space is controlled, a high level of electrical conductivity can be achieved if an efficient electrical pathway is created [41,42]. he formation of an electrical pathway is an important factor in determining how much current will flow through a composite sheet [34]. One way to achieve the formation of an efficient electrical pathway is by interconnecting SWCNTs. Although interconnectivity is closely related to the number of physical contacts between the SWCNTs, this will not be achieved with a high SWCNT content alone. Proper dispersion improves the number of effective physical contacts between the SWCNTs, resulting in more electrical pathways and greater electrical conductivity [41,42]. In this study, the 75_SWCNT/ANF showed electrical conductivity as high as the 100_SWCNT/ANF. In other words, the 75_SWCNT/ANF exhibited a higher level of electrical conductivity even when 25 wt% of the insulator was included. While it is clear that ANFs interfere with the electrical conductivity of the SWCNTs, as ANFs lead to an increase in the number of effective electrical pathways by encouraging the dispersion of SWCNTs [34,41,42,43,44], the electrical conductivity of 75_SWCNT/ANF was as high as that of the 100_SWCNT/ANF.

### 3.4. Joule Heating Effect

**Figure 6:**
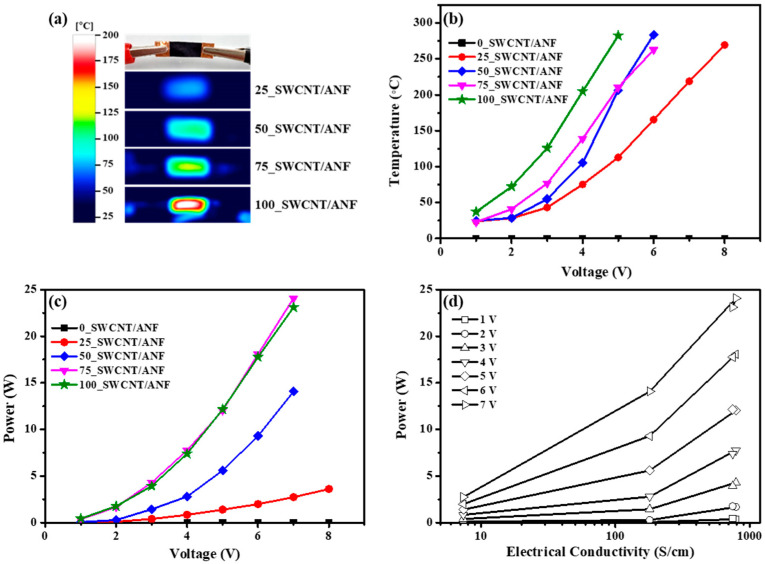
Joule heating effect according to SWCNT content: (**a**) image (thermal imaging camera) of Joule heating system and the resulting temperatures when 4 V was applied, (**b**) temperatures depending on the voltages, (**c**) electric power depending on the input voltages, (**d**) electric power depending on electrical conductivity.

Based on various measurements, a synergistic effect of SWCNTs and ANFs was expected for Joule heating. SWCNTs possess high current density, favorable electrical properties, and high thermal stability, which are important factors that enhance the Joule heating effect [9,10,11]. When SWCNTs reached high temperatures during Joule heating, ANFs, the matrix of this composite, can endure these temperatures due to their high thermal stability. Figure 6 shows the Joule heating effect according to SWCNT content, including (a) an image (thermal imaging camera) of the Joule heating system and the resulting temperatures when 4 V was applied; (b) temperatures depending on the voltages; (c) electric power depending on the input voltages, and (d) electric power depending on electrical conductivity. Figure 6a is an image of the Joule heating system. Samples from each composite sheet, 10 mm in length, 5 mm in width, and 50 μm thick, were heated. The thermal imaging camera showed the heating temperature when 4 V was applied to each sheet. The center point temperatures of each samples were selected as representative heating temperatures. The temperature contour bar indicates 25 to 200 °C. The time to reach equilibrium temperature was 90 s. The images from the thermal imaging camera suggest that the heating temperatures were uniformly distributed when voltage was applied to the composite sheet. The heating temperature data as they varied based on input voltage and SWCNT content are shown in Figure 6b. All samples showed excellent Joule heating behavior, exhibiting over 250 °C despite an input voltage of less than 10 V. The case of 100_SWCNT/ANF showed the highest temperature at the specific input voltage. Increasing the SWCNT content in the composite sheet meant that the number of SWCNTs responding to the input voltage rose. The Joule heating effect was therefore enhanced as the input voltage and SWCNT content increased [28,29,30,31,32,33,34].

Figure 6c is the power depending on the voltage with SWCNT content. The power is expressed as follows:

P = I × V(1)

V = I × R(2)

P = V^2^/R (3)

where P is the power (W) converted from electrical energy to thermal energy, I is current (A), V is voltage (V), and R is resistance (Ω). Equation (2) is Ohm’s law. Based on Equation (3), all plots assessed (Figure 6c) show quadratic curves. As SWCNT content and input voltages were enhanced, electric power sharply improved. Since electrical conductivity (S/cm) includes the multiplicative inverse of resistance (S = 1/Ω), this result is consistent with Equation (3). In particular, the results of 75_SWCNT/ANF and 100_SWCNT/ANF appear similar in Figure 6c, and are consistent with the results of the electrical conductivity tests (Figure 5). Moreover, 75_SWCNT/ANF and 100_SWCNT/ANF exhibited similar electrical conductivity depending on the dispersion of SWCNTs by the ANFs. According to Figure 6c, Equation (3), and the electrical conductivity results, 75 and 100_SWCNT/ANF may require a similar amount of electric power to achieve comparable heating temperatures. Furthermore, electric power as a function of electrical conductivity is shown according to the input voltage in Figure 6d. The higher the electrical conductivity, the more current flowed through each sample based on Equation (1). As the input voltages are enhanced, the allowable electric power increases depending on the electrical conductivity. Ultimately, the sheet heater can therefore be designed to enable efficient energy conversion from electrical energy to thermal energy, accounting for the allowable electric power, input voltage, the electrical conductivity of the composite material, and the heating temperature during Joule heating.

Table 1 shows the heating temperature according to the input voltage by Joule heating compared to previously reported results. Deshpande et al. obtained a spatial temperature of 5 μm long SWCNTs of approximately 400 °C when 1.2 V was applied [11]. A number of other studies have been conducted with various polymer matrixes, filler content, input voltages, electrical properties, etc. Compared to these previously reported results, in our experiment, a large amount of SWCNTs were efficiently dispersed by ANFs to improve the electrical conductivity. Therefore, our developed sheet heater may achieve higher heating temperatures at lower input voltages than the heaters developed in previous studies [28,29,30,31,32,33,34].

### 3.5. Thermo-Mechanical Properties

The 100_SWCNT/ANF consisted of SWCNTs randomly entangled by weak van der Waals forces. These weak interfacial surface interconnections among SWCNTs have yielded disappointing mechanical properties in the past. Therefore, ANFs were carefully selected to provide mechanical properties, as well as to withstand the heating temperature of SWCNTs by Joule heating. SWCNT/ANF composite sheets exhibited a superior Joule heating effect in this study (more than 280 °C). Based on the temperature achieved during Joule heating, the mechanical property was measured by DMA at a temperature range from room temperature to 300 °C. DMA is often used to detect mechanical properties when an external force is applied as a sine wave to specimens as a function of the temperature. Based on the results of TGA and Joule heating behavior, measurement conditions were determined to be 50 to 300 °C, frequency of 1 Hz, and strain of 0.2%.

**Figure 7:**
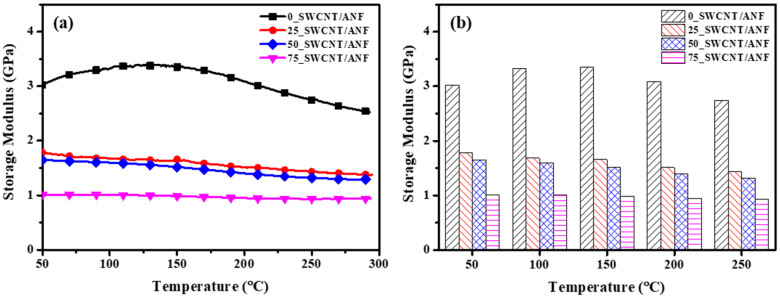
Storage moduli from DMA measurement depending on the temperature. (**a**) Results of storage modulus according to SWCNT content, (**b**) comparison at specific temperatures.

Storage modulus—a measure of the elasticity—was selected from the DMA results to analyze the mechanical properties as a function of the temperature range. The 100_SWCNT/ANF could not be measured by DMA due to its insufficient mechanical property. The storage modulus results of the 0, 25, 50, and 75_SWCNT/ANF are shown in Figure 7. The 0_SWCNT/ANF showed the highest value of around 3 GPa. As the amount of SWCNT increased, the storage modulus decreased. The 75_SWCNT/ANF had the lowest value of around 1 GPa, though this was not a disappointing result in light of the results from other studies [47,48,49,50,51,52]. Furthermore, these results were sustained up to 300 °C. For additional details, Figure 7b shows the storage modulus according to a specific temperature as a bar graph. The mechanical property was maintained up to 300 °C due to the excellent thermal stability of SWCNTs and ANFs. Therefore, the SWCNT/ANF composite sheets maintained their mechanical properties even at the high temperatures reached by Joule heating.

**Figure 8:**
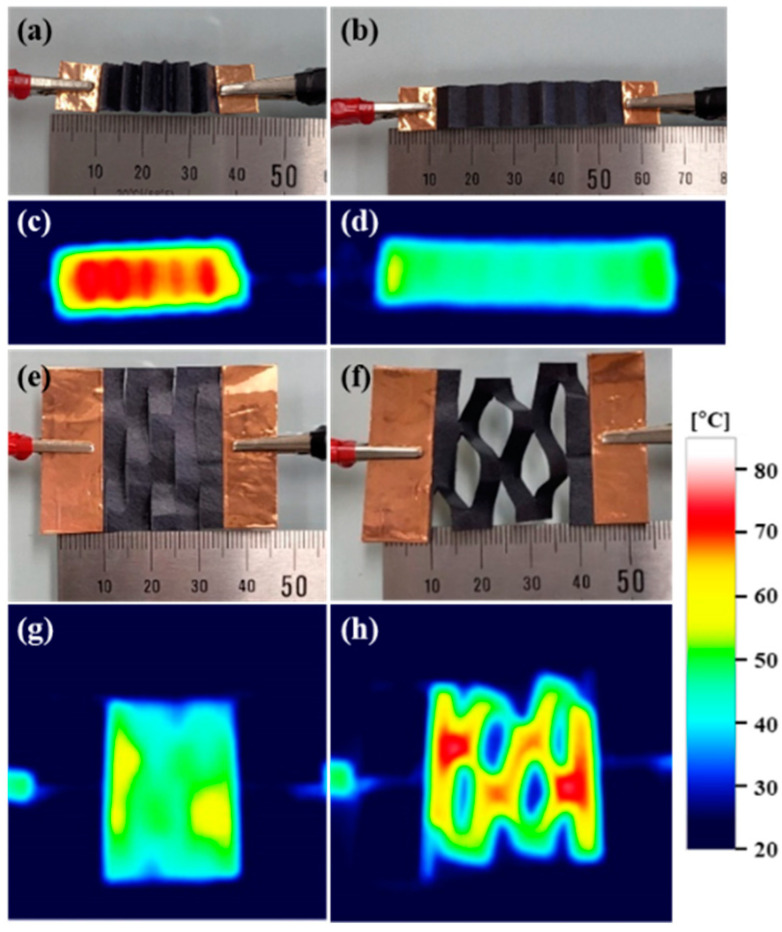
Image of stretchable structure under a Joule heating system. (**a**) Folding state, (**b**) unfolding state, (**e**) Kirigami patterning achieved by a simple cutting method, (**f**) stretching state by the Kirigami structure. Investigation of thermal distribution by thermal imaging camera of (**c**) folding state, (**d**) unfolding state, (**g**) Kirigami pattern, (**h**) stretching state of the Kirigami structure.

Based on the excellent Joule heating effects observed, as well as the thermal stability and mechanical properties of SWCNT/ANF composite sheets, we created a foldable and cuttable sheet heater (Figure 8). Stretchable structures, which are foldable and cuttable, suggest a higher level of possibility than those of flexible and twisted ones. We successfully fabricated a stretchable sheet heater composed of 50_SWCNT/ANF sheets using these folding and cutting characteristics. The temperature contour bar indicates from 20 to 85 °C. Figure 8a is a folded 35 mm, and (b) is an unfolded 55 mm. Electric power was equal to 2.5 W (5 V, 0.5 A), and the temperature changed from Figure 8c,d. In addition, a sheet heater was designed with Kirigami patterning. Typically, complex structures are created through complicated processes such as molding, 3D printing, or cutting by machines, etc. [51], but, in this study, the Kirigami pattern was cut using only a knife. The cutting state is depicted in Figure 8e and the stretching state in Figure 8f. Electric power was maintained up to 2.5 W (5 V, 0.5 A) and there also existed only temperature variation from Figure 8g,h. In short, a new stretchable sheet heater was created, capable of being folded and cut into various shapes. SWCNT/ANF composite sheets have potential for use in a variety of practical de-icing, heating, anti-freezing, wearable, and portable devices employed in the aerospace, automobile, and ship-building industries.

## 4. Discussion

Aramid fiber is a representative polymer with excellent thermal stability that can withstand high temperatures up to around 500 °C [25,26,27]. ANFs made from decomposed aramid fibers have a 1D structure similar to that of SWCNTs. During decomposition, the number of functional groups, including carboxylic acid, carbonyl, and hydroxyl groups, on the surface of the ANF increases [38]. Therefore, we prepared a composite sheet in anticipation of achieving a synergistic effect between SWCNTs and ANFs. As a result of observing the surface and cross-sections of our samples with FE-SEM, small voids were more reduced by ANFs in the 50_SWCNT/ANF than in the 100_SWCNT/ANF. Furthermore, the high temperatures reached during Joule heating were uniformly distributed in each sample. This confirmed that SWCNTs were properly dispersed across the ANF sheet.

The heating temperature reached during Joule heating was measured as a function of SWCNT content and input voltage. As the SWCNT content and input voltage increased, the heating temperature rose. It was established that high electrical conductivity is a reliable indicator of high temperatures at a low input voltage. As a result, we could analyze and evaluate the relationship between allowable electric power, input voltage, electrical conductivity, and heating temperature. The maximum heating temperature reached during Joule heating was reached at around 280 °C in this study. The mechanical properties of SWCNT/ANF composite samples were maintained up to 300 °C.

Based on the excellent Joule heating effect, thermal stability, and mechanical properties, a foldable and cuttable composite sheet was fabricated to suggest a higher level of possibility than those of flexible and twisted ones. Complex structures, such as the Kirigami pattern, were simply cut using a knife instead of a complicated process.

## 5. Conclusions

SWCNT/ANF composites were fabricated and analyzed to evaluate whether they can be used as sheet heaters. We conducted tests of their thermal stability, electrical conductivity, heating temperature (by Joule heating), and mechanical properties. The SWCNTs were successfully dispersed in ANF/DI water, allowing us to obtain SWCNT/ANF composite sheets. TGA showed that our sheet had high thermal stability in an air condition up to around 500 °C. The electrical conductivity of the composite sheet was improved as the amount of SWCNTs added rose to 790.0 and 747.5 S/cm in the 75 and 100_SWCNTs/ANF, respectively. We observed that the Joule heating effect was enhanced as the SWCNT content and input voltage increased. As the SWCNT content and input voltage grew, the electric power quadratically improved. The DMA results showed that the mechanical properties of each sample were maintained up to 300 °C. Ultimately, we successfully obtained a foldable and cuttable composite sheet capable of being formed into a variety of shapes for use as a sheet heater using an uncomplicated fabrication process.

## Figures and Tables

**Table 1:** Heating temperature and input voltage of our nano-filler-based composite sheet compared with those previously reported.

Filler	Concentration (wt%)	Polymer	Voltage (V)	Temperature (°C)	Electrical Conductivity (S/cm)	Resistivity (Ω∙cm)	Reference
SWCNTs	75	ANFs	6	280	7.90 × 10^2^	1.27 × 10^−3^	in this work
	100	ANFs	5	280	7.48 × 10^2^	1.34 × 10^−3^	
Single SWCNT	-	-	1.2	400	1.00 × 10^5^	1.00 × 10^−5^	[11,45]
MWCNTs	10	*m*-aramid	12	270	1.00 × 10^−1^	1.00 × 10^1^	[28]
CNTs	40	^1^ PMIA	15	270	8.00 × 10^1^	1.25 × 10^−2^	[29]
Carboxylic CNTs	30	^1^ PMIA	25	242	1.50 × 10^1^	6.67 × 10^−2^	[30]
CNTs	100	-	2.5	200	1.20 × 10^3^	8.33 × 10^−4^	[31]
MWCNTs/ Graphenes (9/1)	1	^2^ PDMS	50	225	1.37 × 10^−2^	7.30 × 10^1^	[32]
MWCNTs	10	^3^ PBI	25	220	1.00	1.00	[33]
Graphenes	10	epoxy	30	120	1.00 × 10^3^	1.00 × 10^3^	[34]
CNTs	40	ANFs	10	110	2	0.5	[46]

^1^ PMIA: poly (m-phenylene isophthalamide), ^2^ PDMS: polydimethylsiloxane, ^3^ PBI: polybenzimidazole.

## Data Availability

The data presented in this study are available on request from the corresponding author.

## References

[1] Kinloch I.A., Suhr J., Lou J., Young R.J., Ajayan P.M. (2018). Composites with carbon nanotubes and graphene: An outlook. Science.

[2] Lekawa-Raus A., Patmore J., Kurzepa L., Bulmer J., Koziol K. (2014). Electrical Properties of Carbon Nanotube Based Fibers and Their Future Use in Electrical Wiring. Adv. Funct. Mater..

[3] Chiodarelli N., Masahito S., Kashiwagi Y., Li Y.L., Arstila K., Richard O., Cott D.J., Heyns M., De Gendt S., Groeseneken G. (2011). Measuring the electrical resistivity and contact resistance of vertical carbon nanotube bundles for application as interconnects. Nanotechnology.

[4] Kim P., Shi L., Majumdar A., McEuen P.L. (2001). Thermal transport measurements of individual multiwalled nanotubes. Phys. Rev. Lett..

[5] Yu M.F., Lourie O., Dyer M.J., Moloni K., Kelly T.F., Ruoff R.S. (2000). Strength and breaking mechanism of multiwalled carbon nanotubes under tensile load. Science.

[6] Thamaraiselvan C., Wang J.B., James D.K., Narkhede P., Singh S.P., Jassby D., Tour J.M., Arnusch C.J. (2020). Laser-induced graphene and carbon nanotubes as conductive carbon-based materials in environmental technology. Mater. Today.

[7] Xiang R., Inoue T., Zheng Y.J., Kumamoto A., Qian Y., Sato Y., Liu M., Tang D.M., Gokhale D., Guo J. (2020). One-dimensional van der Waals heterostructures. Science.

[8] Krasnikov D.V., Gubarev V.V., Novikov I.V., Kondrashov V.A., Starkov A.V., Krivokorytov M.S., Medvedev V.V., Gladush Y.G., Nasibulin A.G. (2022). Renewable single-walled carbon nanotube membranes for extreme ultraviolet pellicle applications. Carbon.

[9] Costa P.M.F.J., Gautam U.K., Bando Y., Golberg D. (2011). Direct imaging of Joule heating dynamics and temperature profiling inside a carbon nanotube interconnect. Nat. Commun..

[10] Wei B.Q., Vajtai R., Ajayan P.M. (2001). Reliability and current carrying capacity of carbon nanotubes. Appl. Phys. Lett..

[11] Deshpande V.V., Hsieh S., Bushmaker A.W., Bockrath M., Cronin S.B. (2009). Spatially Resolved Temperature Measurements of Electrically Heated Carbon Nanotubes. Phys. Rev. Lett..

[12] Yao Z., Kane C.L., Dekker C. (2000). High-field electrical transport in single-wall carbon nanotubes. Phys. Rev. Lett..

[13] Baloch K.H., Voskanian N., Bronsgeest M., Cumings J. (2012). Remote Joule heating by a carbon nanotube. Nat. Nanotechnol..

[14] Rotkin S.V., Perebeinos V., Petrov A.G., Avouris P. (2009). An Essential Mechanism of Heat Dissipation in Carbon Nanotube Electronics. Nano Lett..

[15] Allaoui A., Hoa S.V., Evesque P., Bai J.B. (2009). Electronic transport in carbon nanotube tangles under compression: The role of contact resistance. Scripta Mater..

[16] Collins P.G., Hersam M., Arnold M., Martel R., Avouris P. (2001). Current saturation and electrical breakdown in multiwalled carbon nanotubes. Phys. Rev. Lett..

[17] Hada M., Hasegawa T., Inoue H., Takagi M., Omoto K., Chujo D., Iemoto S., Kuroda T., Morimoto T., Hayashi T. (2019). One-minute Joule annealing enhances the thermoelectric properties of carbon nanotube yarns via the formation of graphene at the interface. ACS Appl. Energy Mater..

[18] Romanov S.A., Alekseeva A.A., Khabushev E.M., Krasnikov D.V., Nasibulin A.G. (2020). Rapid, efficient, and non-destructive purification of single-walled carbon nanotube films from metallic impurities by Joule heating. Carbon.

[19] Nasibulin A.G., Kaskela A., Mustonen K., Anisimov A.S., Ruiz V., Kivisto S., Rackauskas S., Timmermans M.Y., Pudas M., Aitchison B. (2011). Multifunctional free-standing single-walled carbon nanotube films. ACS Nano.

[20] Amatore C., Berthou M., Hebert S. (1998). Fundamental principles of electrochemical ohmic heating of solutions. J. Electroanal. Chem..

[21] Faruk M.O., Ahmed A., Jalil M.A., Islam M.T., Shamim A., Adak B., Hossain M.M., Mukhopadhyay S. (2021). Functional textiles and composite based wearable thermal devices for Joule heating: Progress and perspectives. Appl. Mater. Today.

[22] Park J. (2020). Functional Fibers, Composites and Textiles Utilizing Photothermal and Joule Heating. Polymers.

[23] Tu K.N., Liu Y.X., Li M.L. (2017). Effect of Joule heating and current crowding on electromigration in mobile technology. Appl. Phys. Rev..

[24] Santini C.A., Vereecken P.M., Volodin A., Groeseneken G., De Gendt S., Van Haesendonck C. (2011). A study of Joule heating-induced breakdown of carbon nanotube interconnects. Nanotechnology.

[25] Bourbigot S., Flambard X. (2002). Heat resistance and flammability of high performance fibres: A review. Fire Mater..

[26] Gore P.M., Kandasubramanian B. (2018). Functionalized Aramid Fibers and Composites for Protective Applications: A Review. Ind. Eng. Chem. Res..

[27] Zhang B., Jia L.H., Tian M., Ning N.Y., Zhang L.Q., Wang W.C. (2021). Surface and interface modification of aramid fiber and its reinforcement for polymer composites: A review. Eur. Polym. J..

[28] Jeong Y.G., Jeon G.W. (2013). Microstructure and Performance of Multiwalled Carbon Nanotube/m-Aramid Composite Films as Electric Heating Elements. ACS Appl. Mater. Interfaces.

[29] Yang B., Ding X.Y., Zhang M.Y., Wang L. (2021). Scalable electric heating paper based on CNT/Aramid fiber with superior mechanical and electric heating properties. Compos. Part B Eng..

[30] Wang L., Zhang M.Y., Yang B., Ding X.Y., Tan J.J., Song S.X., Nie J.Y. (2021). Flexible, Robust, and Durable Aramid Fiber/CNT Composite Paper as a Multifunctional Sensor for Wearable Applications. ACS Appl. Mater. Interfaces.

[31] Aouraghe M.A., Xu F.J., Liu X.H., Qiu Y.P. (2019). Flexible, quickly responsive and highly efficient E-heating carbon nanotube film. Compos. Sci. Technol..

[32] Yan J., Jeong Y.G. (2015). Synergistic effect of hybrid carbon fillers on electric heating behavior of flexible polydimethylsiloxane-based composite films. Compos. Sci. Technol..

[33] Park J., Jeong Y.G. (2015). Investigation of microstructure and electric heating behavior of hybrid polymer composite films based on thermally stable polybenzimidazole and multiwalled carbon nanotube. Polymer.

[34] An J.E., Jeong Y.G. (2013). Structure and electric heating performance of graphene/epoxy composite films. Eur. Polym. J..

[35] Yang M., Cao K.Q., Sui L., Qi Y., Zhu J., Waas A., Arruda E.M., Kieffer J., Thouless M.D., Kotov N.A. (2011). Dispersions of Aramid Nanofibers: A New Nanoscale Building Block. ACS Nano.

[36] Zhu J.Q., Cao W.X., Yue M.L., Hou Y., Han J.C., Yang M. (2015). Strong and Stiff Aramid Nanofiber/Carbon Nanotube Nanocomposites. ACS Nano.

[37] Fan J.C., Shi Z.X., Zhang L., Wang J.L., Yin J. (2012). Aramid nanofiber-functionalized graphene nanosheets for polymer reinforcement. Nanoscale.

[38] Lin J.J., Bang S.H., Malakooti M.H., Sodano H.A. (2017). Isolation of Aramid Nanofibers for High Strength and Toughness Polymer Nanocomposites. ACS Appl. Mater. Interfaces.

[39] Xie F., Jia F.F., Zhuo L.H., Lu Z.Q., Si L.M., Huang J.Z., Zhang M.Y., Ma Q. (2019). Ultrathin MXene/aramid nanofiber composite paper with excellent mechanical properties for efficient electromagnetic interference shielding. Nanoscale.

[40] Fan J.C., Shi Z.X., Tian M., Yin J. (2013). Graphene-aramid nanofiber nanocomposite paper with high mechanical and electrical performance. Rsc. Adv..

[41] Koo M.Y., Shin H.C., Suhr J., Lee G.W. (2021). A Suggested Vacuum Bagging Process for the Fabrication of Single-Walled Carbon Nanotube/Epoxy Composites That Maximize Electromagnetic Interference Shielding Effectiveness. Polymers.

[42] Costa P., Silvia C., Viana J.C., Mendez S.L. (2014). Extruded thermoplastic elastomers styrene-butadiene-styrene/carbon nanotubes composites for strain sensor applications. Compos. Part B Eng..

[43] Drozdov A.D. (2006). A model for the mechanical response of composites with thermoplastic-elastomer matrices. Compos. Sci. Technol..

[44] Theodosiou T.C., Saravanos D.A. (2010). Numerical investigation of mechanisms affecting the piezoresistive properties of CNT-doped polymers using multi-scale models. Compos. Sci. Technol..

[45] Purewal M.S., Hong B.H., Ravi A., Chandra B., Hone J., Kim P. (2007). Scaling of resistance and electron mean free path of single-walled carbon nanotubes. Phys. Rev. Lett..

[46] Hu P.Y., Lyu J., Fu C., Gong W.B., Liao J.H., Lu W.B., Chen Y.P., Zhang X.T. (2020). Multifunctional Aramid Nanofiber/Carbon Nanotube Hybrid Aerogel Films. ACS Nano.

[47] Guo H., Sreekumar T.V., Liu T., Minus M., Kumar S. (2005). Structure and properties of polyacrylonitrile/single wall carbon nanotube composite films. Polymer.

[48] Cheng Q.F., Wang B., Zhang C., Liang Z.Y. (2010). Functionalized Carbon-Nanotube Sheet/Bismaleimide Nanocomposites: Mechanical and Electrical Performance beyond Carbon-Fiber Composites. Small.

[49] Obradovic V., Stojanovic D.B., Jokic B., Zrilic M., Radojevic V., Uskokovic P.S., Aleksic R. (2017). Nanomechanical and anti-stabbing properties of Kolon fabric composites reinforced with hybrid nanoparticles. Compos. Part B Eng..

[50] Shiju J., Al-Sagheer F., Bumajdad A., Ahmad Z. (2018). In-Situ Preparation of Aramid-Multiwalled CNT Nano-Composites: Morphology, Thermal Mechanical and Electric Properties. Nanomaterials.

[51] Bisht A., Dasgupta K., Lahiri D. (2018). Effect of graphene and CNT reinforcement on mechanical and thermomechanical behavior of epoxyA comparative study. J. Appl. Polym. Sci..

[52] Jang N.S., Kim K.H., Ha S.H., Jung S.H., Lee H.M., Kim J.M. (2017). Simple Approach to High-Performance Stretchable Heaters Based on Kirigami Patterning of Conductive Paper for Wearable Thermotherapy Applications. ACS Appl. Mater. Interfaces.

